# Predictive value of pigment epithelial detachment markers for visual acuity outcomes in neovascular age-related macular degeneration

**DOI:** 10.1186/s12886-023-02797-5

**Published:** 2023-03-03

**Authors:** Yiyang Shu, Fuxiang Ye, Haiyun Liu, Jin Wei, Xiaodong Sun

**Affiliations:** 1 Department of Ophthalmology, Shanghai General Hospital, Shanghai Jiao Tong University School of Medicine, Shanghai, 200080 China; 2 National Clinical Research Center for Eye Diseases, Shanghai, China; 3 Shanghai Key Laboratory of Ocular Fundus Disease, Shanghai, China; 4 Shanghai Engineering Center for Visual Science and Photomedicine, Shanghai, China; 5 Shanghai Engineering Center for Precise Diagnosis and Treatment of Eye Diseases, Shanghai, China; 6 Department of Ophthalmology, Shanghai Ninth People’s Hospital, Shanghai Jiao Tong University School of Medicine, Shanghai, China

**Keywords:** Age-related macular degeneration, Polypoidal choroidal vasculopathy, Pigment epithelial detachment, Optical coherence tomography, Images marker

## Abstract

**Background:**

To determine the predictive value of quantitative morphological parameters for pigment epithelial detachment (PED) of neovascular age-related macular degeneration (nAMD) patients.

**Methods:**

One eye from each of 159 patients with nAMD were studied. Polypoidal choroidal vasculopathy (PCV) group included 77 eyes, and non-PCV group 82. Patients received conbercept 0.05 ml (0.5 mg) in a 3 + ProReNata (PRN) treatment regimen. Correlations between retinal morphologic parameters at baseline and best-corrected visual acuity (BCVA) gain at 3 or 12 months after treatment (structure–function correlations) were assessed. Optical coherence tomography (OCT) scans were used to assess retinal morphologic features including intraretinal cystoid fluid (IRC), subretinal fluid (SRF), PED or PED type (PEDT), and vitreomacular adhesion (VMA). Greatest height (PEDH) and width of PED (PEDW), and volume of PED (PEDV) at baseline were also measured.

**Results:**

For non-PCV group, BCVA gain from 3 or 12 months after treatment was negatively correlated with PEDV at baseline (*r* = -0.329, -0.312, *P* = 0.027, 0.037). BCVA gain at 12 months after treatment was negatively correlated with PEDW at baseline (*r* = -0.305, *P* = 0.044). For PCV group, there were no correlations with PEDV, PEDH, PEDW, and PEDT in BCVA gain between baseline and 3 or 12 months after treatment (*P* > 0.05). SRF, IRC, VMA at baseline did not correlate with short-term and long-term BCVA gain in patients with nAMD (*P* > 0.05).

**Conclusion:**

For patients with non-PCV, PEDV at baseline was negatively correlated with short-term and long-term BCVA gain, and PEDW was negatively correlated with long-term BCVA gain. On the contrary, quantitative morphological parameters for PED at baseline had no correlation with BCVA gain in patients with PCV.

**Supplementary Information:**

The online version contains supplementary material available at 10.1186/s12886-023-02797-5.

## Introduction

Conbercept as a recommended anti-vascular endothelial growth factor (VEGF) fusion protein, has been developed and approved [1]. A phase III randomized trial has been confirmed that conbercept can improve visual acuity in patients with neovascular age-related macular degeneration **(**nAMD) [2]. Studies have also shown conbercept to effectively improve visual acuity of patients with polypoidal choroidal vasculopathy (PCV) [3, 4].

Optical coherence tomography (OCT) paired with quantitative analysis, has been widely used for qualitative assessment of different retinal compartments, including intraretinal, subretinal, and subpigment epithelial [5]. For patients with nAMD, such quantitative analysis may reveal functionally relevant microstructural changes not captured by measurements of the retina. These include intraretinal fluid (IRF), pigment epithelial detachment (PED), subretinal fluid (SRF), and vitreomacular adhesion (VMA). Some retinal morphologic parameters have already been identified to predict treatment responses; however, the effect of some morphologic parameters on visual acuity in AMD patients remains controversial. The significance of PED presence as a prognostic indicator for poor visual outcome in patients with nAMD on anti-VEGF treatment has been highlighted [6–8], whereas evidence suggests that SRF is not associated with vision decline [9–12]. In addition, there are reports that the presence of IRC may lead to visual loss [9], and that VMA is not associated with visual acuity [13].

In this study, the qualitative and quantitative analysis of PED, such as the greatest height (PEDH) and width of PED (PEDW), volume of PED (PEDV) and PED type (PEDT) at baseline for AMD patients was conducted to identify imaging markers for patients with different best-corrected visual acuity (BCVA) gain at 3 or 12 months after treatment. nAMD patients were then further divided into PCV group and non-PCV group to explore whether PCV had different effects on BCVA gain. This will aid in identifying imaging markers related to the visual prognosis for PCV and non-PCV groups, and guide investigations into related mechanisms. In addition, we also investigated the relationship between other baselines for OCT morphologic parameters and BCVA gain, including SRD, IRC, VMA.

## Methods

### Study participants

The study protocol was approved by the Ethics Committees of Shanghai General Hospital affiliated with Shanghai Jiaotong University School of Medicine. All human participants have given written informed consent.

In this retrospective study, nAMD patients were all treated with intravitreal injections of conbercept (0.5 mg/0.05 mL) following a 3 + ProReNata (PRN) treatment regimen for at least 12 months at the Department of Ophthalmology, Shanghai General Hospital, Shanghai Jiao Tong University School of Medicine, from March 2017 to July 2019. Diagnosis of nAMD was performed on fluorescein angiography (FA) and indocyanine green angiography (ICGA). Exclusion criteria were ocular trauma, or any associated concurrent eye conditions such as high myopia, glaucoma, or uveitis and history of previous intravitreal injection.

All nAMD patients underwent a complete ophthalmologic examination, which included measurements of BCVA, intraocular pressure, fundus examination, color fundus photography, FA, ICGA, or OCTA. BCVA testing was performed using early treatment of diabetic retinopathy study (ETDRS) charts with an initial testing distance of 4 m, completed by one experienced tester after standardized refraction. The main outcomes measured were the BCVA gain between baseline to 3 and 12 months after treatment. A complete and standardized eye examination including BCVA and OCT was measured at each monthly visit. Only one eye of each patient was selected for the study. After initial screening, patients with PCV were identified for grouping purposes.

### Morphologic analysis with optical coherence tomography

**Fig. 1:**
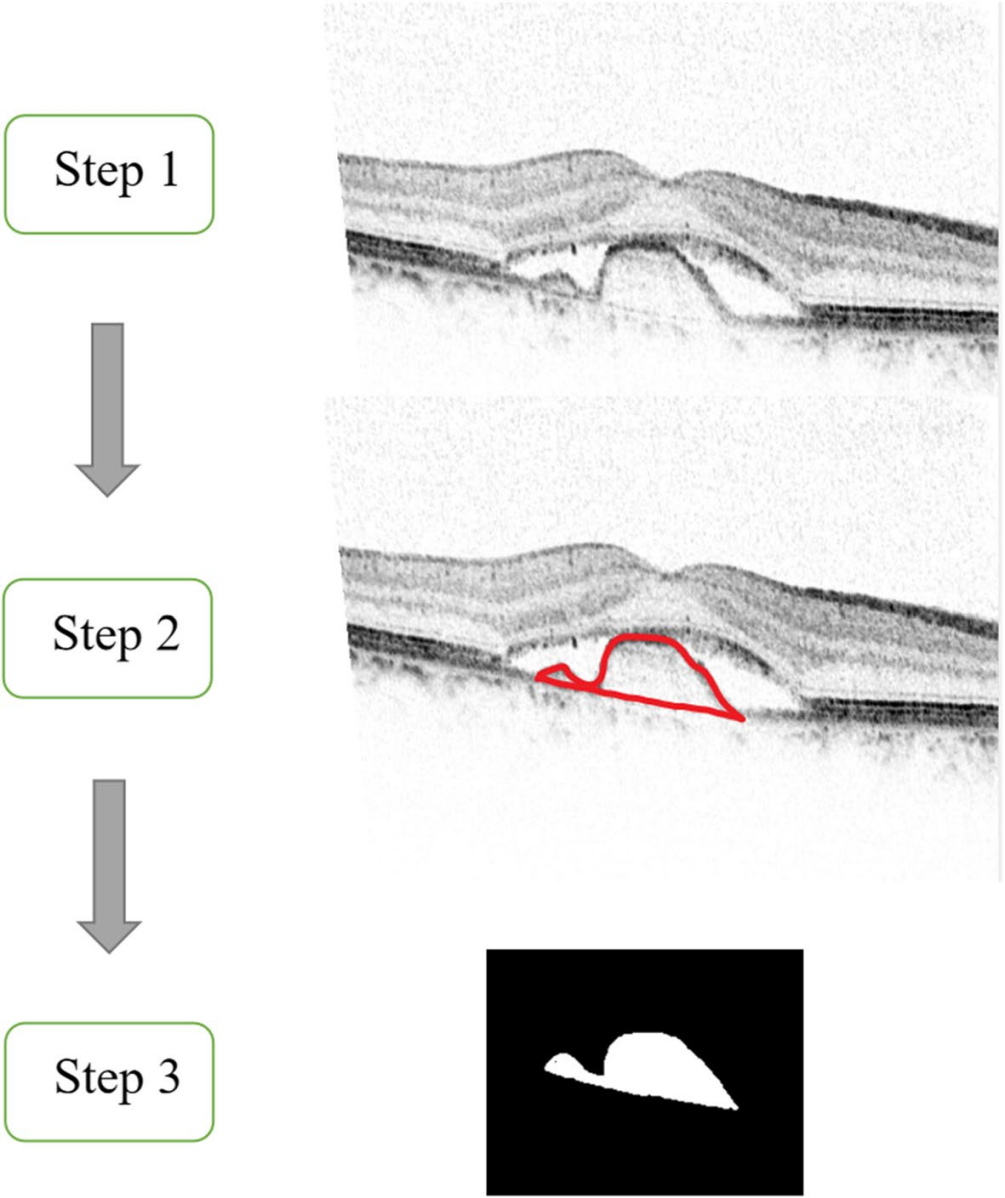
Flow chart of measuring PEDV using ImageJ. Step 1, take out the PED scan that needs to be measured; Step 2, circle the outline of the PED; Step 3, measure the circled area. This is the PED area of a B-scan. The summation of the whole PED area in each volume scan was used as an index for PEDV. *PEDV* the volume of PED

All OCT images were obtained by the Heidelberg Spectralis spectral domain OCT (SD-OCT) (Heidelberg Engineering, Heidelberg, Germany). The following scan patterns were performed on both eyes and centered on the fovea: 20 × 15 degree raster scans consisting of 19 high-speed line scans. IRF, SRF, PED, VMA, and PEDT were determined from the baseline OCT images and PEDH and PEDW at baseline were measured by two ophthalmologists blinded to the group assignments. The distance was taken between the inner surface of the Bruch’s membrane and the outer surface of the RPE. The PED area of each B-scan was measured using ImageJ (an open-source software developed by Wayne Rasband, National Institutes of Health, Bethesda, MD, USA). The summation of the whole PED area in each volume scan was used as an index for PEDV (Fig. 1). The two ophthalmologists performed triplicate measurements respectively and recorded the average value. PED was not counted if there was not at the fovea.

**Fig. 2:**
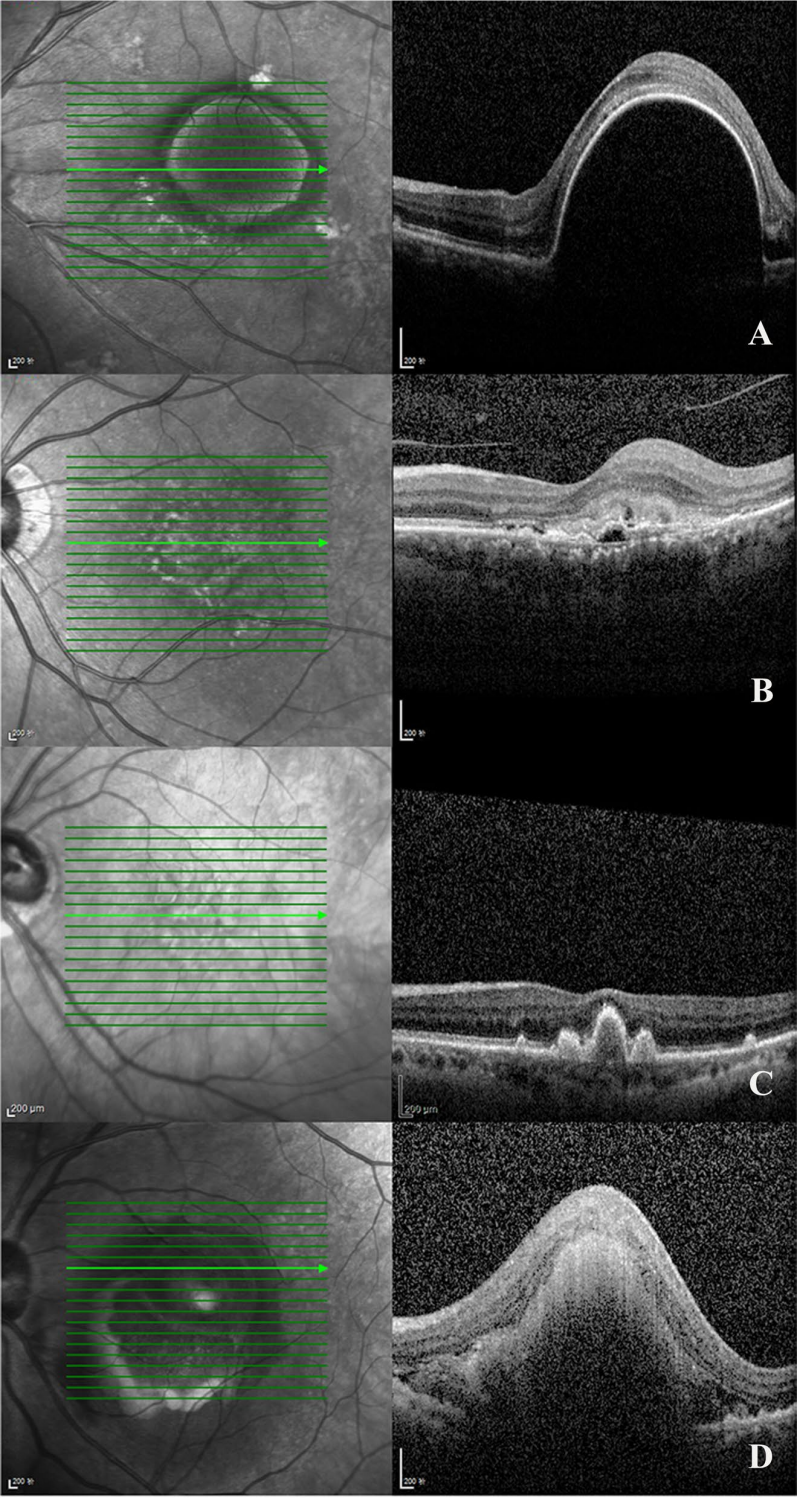
Imaging SD-OCT. **A** Serous PED; **B** Fibrovascular PED; **C** Drusenoid PED; **D** Hemorrhagic PED. *PED* pigment epithelial detachment

PED is a prominent feature of the AMD process, manifesting as an anatomical separation of retinal pigment epithelium (RPE) from the underlying Bruch layer [14]. PEDs were classified as either serous, fibrovascular, drusenoid, or hemorrhagic (Fig. 2). Drusenoid PEDs are composed of one or more drusen or soft drusen. Serous PEDs usually consists mainly of hyporeflective material. Fibrovascular PEDs can be seen as a hyperreflective material adhered to the outer PED layer. Hemorrhagic PEDs show a mixture of hyporeflective and hyperreflective material [15].

### Statistical analysis

Statistical analysis was performed using IBM SPSS Statistic 24. Qualitative variables were described in percentages and quantitative variables were described as mean ± standard deviation. Paired t-test was performed to compare BCVA before and after treatment. Students' t test and Mann–Whitney U test for continuous variables was used for statistical analysis between 2 groups. χ^2^ test for categorical variables was used for statistical analysis between 2 groups. Spearman rank correlation was used to analyze the correlation between PED at baseline and BCVA gain. *P* < 0.05 was considered significant.

## Results

### Clinical characteristics

A total of 159 eyes of 159 AMD patients were enrolled for the study. The eyes were classified into PCV group and non-PCV group. 77 eyes of 77 patients (50 male and 27 female) were in the PCV group, and 82 (42 male and 40 female) in the non-PCV group. The mean BCVA in PCV group was 48.78 ± 19.92, and for the non-PCV group, 48.12 ± 16.75. Follow-up was at least 12 months. For the non-PCV group, the presence of SRF was noted in 59 eyes, IRC in 28 eyes, PED in 45 eyes, and VMA in 20 eyes at baseline. These patients had a mean of 4.43 ± 2.29 anti-VEGF injections at follow-up. For the PCV group, 65 eyes had SRF, 18 had IRC, 59 had PED, and 17 had VMA at baseline. These patients had a mean of 5.71 ± 1.90 anti-VEGF injections at follow-up. The clinical characteristics of patients in each group are summarized in Table 1.

**Table 1:** Clinical characteristics of patients with age-related macular degeneration managed with a 3 + PRN protocol

	PCV group (*n* = 77)	Non-PCV group (*n* = 82)	*P*
**Baseline Characteristics**			
Age, years	65.81 ± 9.63	68.70 ± 8.31	0.155
Sex (Male/Female)	50/27	42/40	0.080
BCVA (ETDRS letters at first visit)	48.78 ± 19.92	48.12 ± 16.75	0.822
SRF (No/Yes)	12/65	23/59	0.058
IRC (No/Yes)	59/18	54/28	0.182
PED (No/Yes)	18/59	37/45	0.011*
VMA (No/Yes)	60/17	62/20	0.985
Anti-VEGF injection, number	5.71 ± 1.90	4.43 ± 2.29	< 0.001*
**Follow up data**			
BCVA gain at 3 M	6.34 ± 14.64	9.91 ± 15.66	0.102
BCVA gain at 12 M	4.42 ± 20.83	9.37 ± 17.05	0.206

*SRF* Subretinal fluid, *IRC* Inner retinal cyst, *PED* Pigment epithelial detachment, *VMA* Vitreomacular adhesion, *PCV* Polypoidal choroidal vasculopathy, *VEGF* Vascular endothelial growth factor, *BCVA* Best-corrected visual acuity, *BCVA gain at 3 M* BCVA changes at three months after treatment and baseline, *BCVA gain at 12 M* BCVA changes at twelve months after treatment and baseline, *M* Month

^*^P < 0.05 was set as statistical significance

### BCVA

**Fig. 3:**
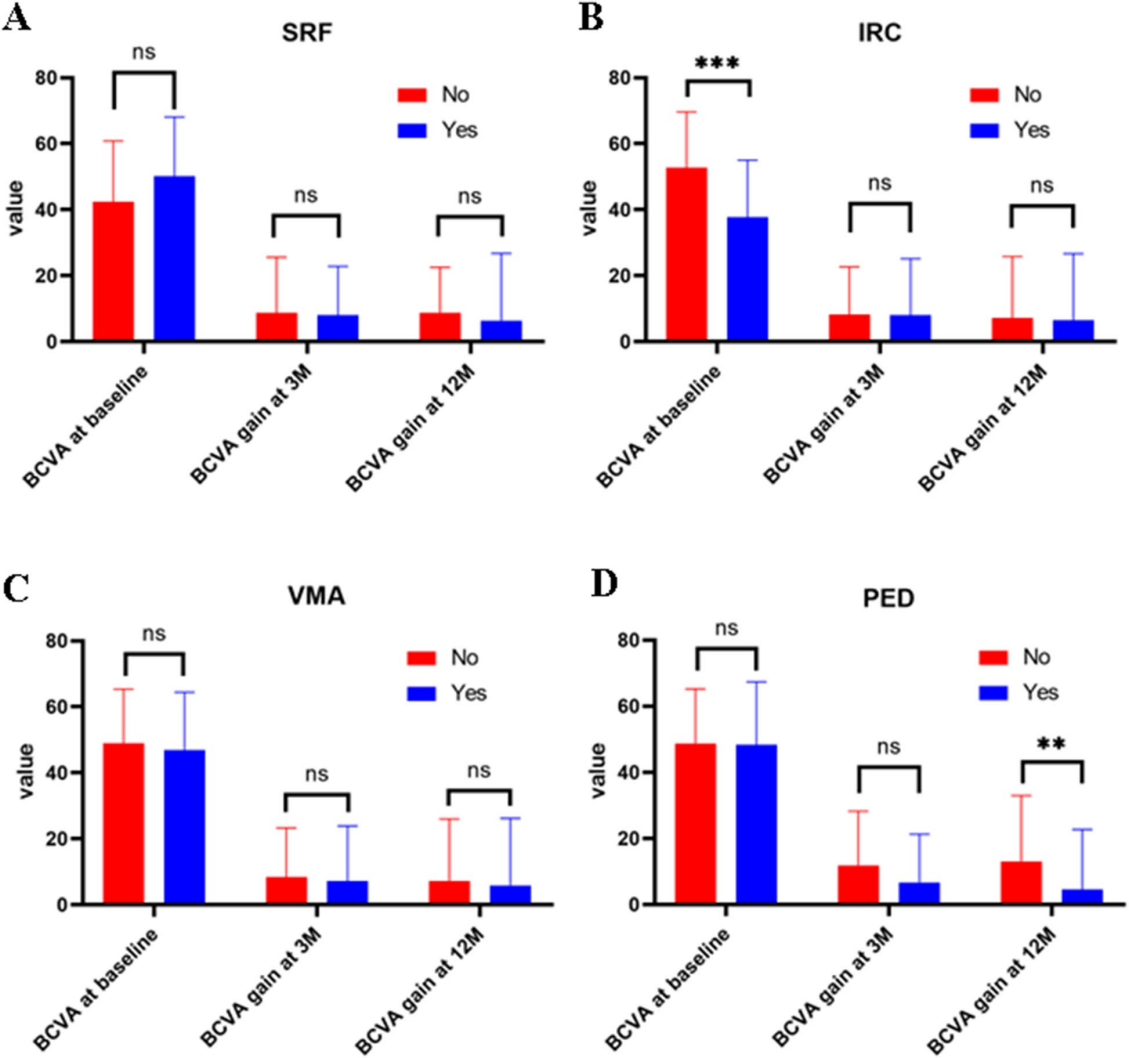
The correlations between baseline retinal morphologic parameters and BCVA at baseline, BCVA gain at 3 or 12 months: **A** SRF; **B** IRC; **C** VMA; **D** PED. *SRF* subretinal fluid, *IRC* inner retinal cyst, *VMA* vitreomacular adhesion

For all eyes (*n* = 159), we compared the BCVA before treatment, BCVA gain at 3 months and 12 months after treatment for patients with or without PED, SRF, IRC, and VMA. The correlations between baseline retinal morphologic parameters and BCVA gain are summarized in Fig. 3 ( Supplementary Table 1). The presence of PED on BCVA and BCVA gain at 3 months and 12 months after treatment in PCV and non-PCV group were then studied. We found BCVA at baseline had no difference in either PCV or non-PCV group with or without PED, and that only BCVA gain in 12 months was negatively correlated with PED in nAMD (*P* = 0.006).

In the non-PCV group (*n* = 82), BCVA at 3 and 12 months after treatment was (58.28 ± 16.76) and (57.49 ± 17.31), respectively. The BCVA gain was (9.91 ± 15.66) and (9.37 ± 17.05), respectively. Compared with BCVA (48.12 ± 16.75) at baseline, there were significant differences by 3 and 12 months (t = 5.662, 4.973, *P* < 0.001). However, there was no significant difference in BCVA between 3 and 12 months after treatment (t = 0.630, *P* = 0.53).

In the PCV group (*n* = 77), BCVA at 3 months, 12 months after treatment BCVA was (55.12 ± 18.64) and (53.19 ± 22.41), and the BCVA gain was (6.34 ± 14,64) and (4.42 ± 20.83). Compared with BCVA (48.78 ± 19.92) at baseline, there was statistically significant difference at 3 month (t = 3.798, *P* < 0.001). However, there was no significant difference in BCVA between baseline and at 12 months, as well as between 3 and 12 months after treatment (t = 1.806,1.104, *P* = 0.067,0.273).

### Correlation analysis

In the non-PCV group (*n* = 45), BCVA gain between the baseline and 3 months after treatment were negatively correlated with PEDV at the baseline (*r* = -0.329, *P* = 0.027). There were no correlations with PEDW, PEDH, and PEDT (*r* = -0.289, -0.134, -0.105, *P* = 0.057, 0.384, 0.494). The BCVA gain at the baseline and 12 months after treatment were negatively correlated with PEDV and PEDW at the baseline (*r* = -0.312, -0.305, *P* = 0.037, 0.044). There was no correlation between PEDH, PEDT and BCVA gain at 3 or 12 months (*r* = -0.231, 0.027, *P* = 0.132, 0.861) (Table 2).

**Table 2:** Correlation of baseline PED characteristics with BCVA gain at 3 and 12 months in non-PCV group (*n* = 45)

	BCVA gain at 3 M	BCVA gain at 12 M
PEDV	*r* = -0.329, *P* = 0.027*	*r* = -0.312, *P* = 0.037*
PEDW	*r* = -0.289, *P* = 0.057	*r* = -0.305, *P* = 0.044*
PEDH	*r* = -0.134, *P* = 0.384	*r* = -0.231, *P* = 0.132
PEDT	*r* = -0.105, *P* = 0.494	*r* = 0.027, *P* = 0.861

*PEDV* the volume of the PED, *PEDW* the maximum base width of PED, *PEDH* the maximum height of PED, *PEDT* the type of PED

^*^Spearman correlations with *P* < 0.05 were deemed significant

In the PCV group (*n* = 59), there were no correlations with PEDV, PEDH, PEDW, and PEDT for BCVA gain between baseline and at 3 or 12 months after treatment (*P* > 0.05) (Table 3).

**Table 3:** Correlation of baseline PED characteristics with BCVA gain at 3 and 12 months in PCV group (*n* = 59)

	BCVA gain at 3 M	BCVA gain at 12 M
PEDV	*r* = 0.251, *P* = 0.055	*r* = 0.195, *P* = 0.138
PEDW	*r* = 0.143, *P* = 0.281	*r* = 0.123, *P* = 0.355
PEDH	*r* = 0.250, *P* = 0.056	*r* = 0.160, *P* = 0.225
PEDT	*r* = 0.016, *P* = 0.901	*r* = 0.153, *P* = 0.248

## Discussion

We aimed to investigate morphologic biomarkers in OCT to determine visual acuity outcomes for nAMD treated with 3 + PRN conbercept. The results of our study showed that intravitreal conbercept therapy of 3 + PRN could effectively improve the BCVA in patients. Although BCVA saw greater improvements in non-PCV group than in PCV group, there was no significant difference (*p* > 0.05). nAMD patients were grouped according to the morphological characteristics of OCT at baseline. There was no significant difference at baseline BCVA for patients with or without PED at baseline, while there was a difference in BCVA gain at 3 months, but it was not significant. At 12 months, there was a significant difference in BCVA gain, where non-PED group yielded better gains than that of the PED group. IRC had a significant impact on visual acuity. At baseline, the visual acuity of the IRC group was significantly worse than that of the non-IRC group. But with treatment, there was no significant difference between BCVA gain at 3 and 12 months. VMA and SRF had no significant effects on BCVA and BCVA gain.

Although PCV was initially thought to be a choroidal disease, studies [16] have found that PCV was not directly derived from normal choroidal vessels, but originated from type 1 CNV, which may be more accurately referred to as "neovascularization" rather than choroidal disease. Consensus on Neovascular Age-Related Macular Degeneration Nomenclature Study Group in 2020 also classified this as type 1 macular neovascularization (MNV) [17].

PED is a prominent clinical manifestation in multiple disease processes and often contributes to loss of central vision [18]. The BCVA of the two groups were different at baseline, although there were no significant differences. In our study, PEDs were classified as either serous, fibrovascular, drusenoid, or hemorrhagic. Although it may be important to consider PEDT to predict the visual acuity in patients treated by intravitreal injection, the anatomical response of the PED may not correlate directly with the visual outcome [19]. No matter what kind of PED, it causes lesions in the RPE layer, leading to retinal dysfunction, which may cause loss of vision. Serous PED may not be fully absorbed. Hemorrhagic PED is toxic to the retina, thus even if the bleeding is fully absorbed, and the retinal morphology recovers, visual loss may remain. Visual function and morphology have a certain correlation, but other factors also play a role, such as bleeding and interlaminar structure destruction, disease progression, speed of absorption, and disease destruction. PEDT may predict morphologic prognosis but not functional prognosis. Different types of PED present different morphologic prognoses after treatment, and visual improvements are related to a myriad of factors. This could explain why PEDT does not correlate with BCVA gain. In our study, we aimed to explore the indicative effect of PED on the BCVA gain of nAMD patients. Thus, nAMD patients with PED were divided into PCV and non-PCV groups.

Many studies have been focusing on the influence of PED on visual acuity in nAMD which remains controversial. The results we obtained are in accordance with previous studies that suggested presence of PED had a negative correlation with BCVA gain for non-PCV [7, 20–22]. Building upon this finding, we conducted further quantitative assessment, including PEDW, PEDH, PEDV, PEDT at baseline. Correlation analysis was performed with BCVA gain at 3 and 12 months, respectively. The results showed that wider PEDW at baseline correlates to less BCVA gain at 12 months. This is most likely due retinal structure damage caused by the extent of the lesion, which may depend on the duration of the pathology. A wider PED translates to larger and more severe lesion. In the short term, through active treatment, BCVA may see improvement yielding no significant negative correlation between BCVA gain and PEDW. Following disease progresses, some other retinal structural damage, such as atrophy of the RPE may emerge, thus the favorable results observed at 3 months after treatment may not be able to be maintained in the long term, resulting in a poorer prognosis. We also found PEDV at baseline was negatively correlated with BCVA gain at 3 and 12 months in patients with non-PCV. This could be due to the association of PED with CNV, where larger CNV is associated with poorer prognosis for vision. The volume also better reflects the scope and extent of the lesion, which we found to have markedly different influences on BCVA gain.

Data on the effects of PED on specific visual outcomes of PCV are limited and inconsistent. Our results are in accordance with other previously published studies that the presence of PED had no correlation with BCVA gain for PCV [20]. In our study, the BCVA gain in 3 or 12 months in the non-PCV group was greater than that in the PCV group, although there was no significant difference. PEDV at baseline in PCV group was larger than that in non-PCV group, while BCVA gain was relatively smaller, although there was no statistical difference between the two groups, which may explain why PED has an indication effect for non-PCV group but not for PCV group. However, we believe that PEDV at baseline may still affect vision prognosis. PED is not a good evaluation of the visual prognosis for patients with PCV, it may be related to bleeding and serous fluid. After absorption, the vision prognosis may be more positive.

In the clinic, we can easily measure PEDH and PEDW based on the OCT image while the patient has finished the OCT. However, additional software is needed for the measurement of PEDV. With the popularity and development of artificial intelligence (AI), radiomics and other methods, we can work on a predictive model where the parameters of PED are automatically identified by inputting OCT images, and thus output the predicted visual acuity in the future research. This will provide us with a basis for treatment and provide patients with highly reliable prediction results. Our study had certain limitations. Firstly, because of the retrospective study design, there was a possibility of irregular follow-up, and only limited information could be collected. Larger prospective studies covering larger cohorts are needed to validate these results. Secondly, the scan patterns were performed with 20 × 15 degree which may miss the areas between the scans. Finally, when assessing PED volume, manual correction had to be used.

## Conclusion

In conclusion, the aim of this study was to detect the parameters of PED by dividing AMD patients treated with intravitreal injection of conbercept into PCV and non-PCV group. The results confirmed that the PEDV could predict the short- and long-term BCVA gains for non-PCV after intravitreal injection of conbercept. In contrast, quantitative morphological parameters for PED had no predictive effect on the short- and long-term BCVA gains for PCV patients with intravitreal injection of conbercept. Despite the limitations of this retrospective study, our findings raised some interesting questions as the type of PED was found to not affect BCVA gain. The BCVA gain of PCV is less than that of non-PCV, but there was no significant difference in the results. Our study follows similar procedures conducted for other anti-VEGF drugs, thus our results may provide reference values for other anti-VEGF drugs as well. Furthermore, this may have clinical useful implications for predicting the prognosis and monitoring the follow-up of nAMD patients.

## Supplementary Information

**Additional file 1:** **Supplementary Table 1.** The correlations between baseline retinal morphologic parameters and BCVA at baseline, BCVA gain at 3 or 12 months (*n*=159).

## Data Availability

The data used or analyzed during the current study are available from the corresponding author on reasonable request.
