# Supplementary material for: Predictive value of pigment epithelial detachment markers for visual acuity outcomes in neovascular age-related macular degeneration
Source: BMC Ophthalmol. 2023 Mar 3;23:83. doi: 10.1186/s12886-023-02797-5 (PMC9983272; doi:10.1186/s12886-023-02797-5)
Supplement: Supplementary file 1 — Additional file 1: Supplementary Table 1. The correlations between baseline retinal morphologic parameters and BCVA at baseline, BCVA gain at 3 or 12 months (n=159). [file 12886_2023_2797_MOESM1_ESM.doc]

| **Supplementary Table 1** The correlations between baseline retinal morphologic parameters and BCVA at baseline, BCVA gain at 3 or 12 months(n=159). | | | | | | |
| --- | --- | --- | --- | --- | --- | --- |
|  | BCVA at baseline | P | BCVA gain at 3M | P | BCVA gain at 12M | P |
| SRF |  |  |  |  |  |  |
| No(n=35) | 42.43±18.42 |  | 8.76±16.90 |  | 8.82±13.72 |  |
| Yes(n=124) | 50.14±17.98 | 0.056 | 8.00±14.82 | 0.704 | 6.44±20.35 | 0.950 |
| IRC |  |  |  |  |  |  |
| No(n=114) | 52.69±16.93 |  | 8.21±14.49 |  | 7.14±18.70 |  |
| Yes(n=45) | 37.67±17.35 | <0.001^***^ | 8.04±17.10 | 0.948 | 6.53±20.20 | 0.814 |
| PED |  |  |  |  |  |  |
| No(n=46) | 48.78±16.47 |  | 11.93±16.33 |  | 13.11±19.84 |  |
| Yes(n=113) | 48.30±19.06 | 0.991 | 6.69±14.59 | 0.089 | 4.47±18.26 | 0.006^**^ |
| VMA |  |  |  |  |  |  |
| No(n=124) | 48.88±18.56 |  | 8.44±14.83 |  | 7.27±18.78 |  |
| Yes(n=35) | 46.89±17.52 | 0.522 | 7.17±16.74 | 0.871 | 5.91±20.35 | 0.851 |
| *SRF* subretinal fluid, *IRC* inner retinal cyst, *PED* pigment epithelial detachment, *VMA* vitreomacular adhesion, *BCVA* best-corrected visual acuity, *BCVA gain at 3M* BCVA changes at three months after treatment and baseline, *BCVA gain at 12M* BCVA changes at twelve months after treatment and baseline. *M* month  Mann-Whitney U test for continuous variables was used for statistical analysis between 2 groups;  P < 0.05 was set as statistical significance. ^***^p<0.001, ^**^P<0.01, ^*^P<0.05. | | | | | | |
